# Symptom clusters in inflammatory bowel disease: a scoping review

**DOI:** 10.3389/fmed.2025.1615100

**Published:** 2025-09-16

**Authors:** Keying Xu, Mengjiao Li, Ping Jiang

**Affiliations:** ^1^Graduate School, Shanghai University of Traditional Chinese Medicine, Shanghai, China; ^2^Pudong New Area People's Hospital, Shanghai, China

**Keywords:** inflammatory bowel disease, symptom cluster, symptom distress, assessment tool, scoping review

## Abstract

**Background:**

Inflammatory bowel disease (IBD) is a chronic relapsing gastrointestinal disorder with a high symptom burden. Patients often report multiple concurrent symptoms, yet most studies have examined them individually. Symptom clusters—defined as groups of co-occurring and interrelated symptoms—provide a framework for understanding this complexity. Evidence on IBD-specific clusters, however, remains fragmented and inconsistent.

**Methods:**

We conducted a scoping review following Arksey and O'Malley's framework to synthesize findings on IBD symptom cluster types, assessment instruments, associated factors, and dynamic trajectories. Searches covered database inception through 31 October 2024.

**Results:**

Thirteen studies were included, identifying 29 symptom clusters. Marked heterogeneity and overlap were observed. To integrate findings, clusters were categorized into five groups: gastrointestinal/physical, psychological, systemic/fatigue, nutritional/appetite-related, and mixed/trajectory-related. Assessment instruments varied considerably, most lacking IBD-specific validation. Reported associated factors included demographic, clinical, and treatment variables, but results were inconsistent. Few studies addressed longitudinal changes or interactions among clusters.

**Conclusion:**

Research on IBD symptom clusters remains limited and heterogeneous. Standardized definitions and validated tools are urgently needed. Most existing studies did not stratify findings by disease subtype, although limited evidence indicates that UC and CD appear to exhibit distinct clustering patterns. Future studies should adopt longitudinal and biomarker-informed designs, and examine interactions among clusters, to improve clinical management and patient outcomes.

## Introduction

Inflammatory bowel disease (IBD) encompasses ulcerative colitis (UC) and Crohn's disease (CD). It is a chronic and relapsing inflammatory condition of the gastrointestinal tract (1). Although the exact etiology is not fully elucidated, extensive research has established that IBD arises from multifactorial interactions among genetic predisposition, environmental triggers, gut microbiota alterations, and dysregulated immune responses (2–4). Historically, IBD was predominantly reported in Western countries, such as North America, the United Kingdom, and Northern Europe (5, 6). In contrast, with rapid changes in diet and lifestyle, newly industrialized regions such as Asia and Africa are witnessing sharp increases in incidence (7). Given this global trend, IBD has emerged as a major public health challenge (8–12).

Patients with IBD frequently experience multiple concurrent symptoms, with nearly three-quarters reporting two or more at the same time (13, 14). Despite this, most existing studies have focused on single symptoms, overlooking the complexity of co-occurrence. This highlights the need for approaches that consider interrelationships among symptoms rather than isolated phenomena (15). One such approach is the concept of the symptom cluster, introduced by Dodd in 2001 as a grouping of three or more concurrent symptoms, later refined by Kim in 2005 to include two or more correlated symptoms (16, 17). Studying clusters rather than individual symptoms allows for a more holistic understanding of patients' symptom experiences, facilitates targeted interventions, and may ultimately improve quality of life (18–20).

However, current research on IBD symptom clusters remains limited and heterogeneous. The classification of clusters and the instruments used to measure them vary widely, with no consensus across studies. To address this gap, we conducted a scoping review following the methodological framework proposed by Arksey and O'Malley (21). Our objectives were to summarize existing evidence on cluster types, assessment instruments, and dynamic changes, and to provide a reference framework for the standardized management of symptom clusters in IBD.

## Method

### Protocol

To ensure the study's transparency and reproducibility, the methodological framework described by Arksey and O'Malley was used to conduct the scoping review. This framework includes defining the research question, identifying relevant studies, study selection, graphing the data, and collating, summarizing, and reporting the results. Reporting followed the PRISMA-ScR checklist.

### Research question

Define the research questions for the scoping review: (1) What are the types of symptom clusters in patients with IBD? (2) What assessment tools are for symptom clusters in patients with IBD? (3) What factors are associated with symptom clusters in patients with IBD? (4) Do symptom clusters change dynamically in patients with IBD?

### Identifying relevant studies

#### Information sources

The databases examined comprised Web of Science, PubMed, Cochrane Library, EMBASE, CINAHL, CNKI, Wanfang Data, VIP Database, and SinoMed. The most recent search date was 31 October 2024.

#### Search strategy

The nine databases were searched using a combination of Medical Subject Headings (MeSH) and free words. The keywords searched were “Inflammatory Bowel Diseases”, “inflammatory bowel disease”, “bowel diseases inflammatory”, “Crohn's disease”, “ulcerative colitis”, “syndrome”, “symptom cluster”, “symptom combination”, “multiple symptoms”, “symptom constellation”, “concurrent symptom”. The research team began with an initial search in PubMed and CNKI to refine their approach. After carefully reviewing and assessing the results, they optimized their strategy before conducting the formal search.

### Study selection

#### Inclusion and exclusion criteria

Inclusion criteria: (1) the study was conducted in patients with IBD, aged ≥18 years; (2) the study involved symptom clusters or correlations between multiple symptoms; (3) there was no restriction on the type of study design, including quantitative, qualitative, and mixed studies; and exclusion criteria were: (1) duplicate publications or unavailability of the full text; (2) non-Chinese and English literature; (3) reviews, guidelines, conference papers, and opinions.

#### Screening process

The extracted article titles were imported into the Endnote program to eliminate duplication. Two researchers independently evaluated the titles and abstracts based on the inclusion and exclusion criteria. The articles that satisfied the inclusion criteria were imported with their full-text attachments for thorough examination.

### Charting the data

Two researchers independently extracted data and information and cross-checked their findings. A third reviewer resolved disagreements. The extracted data included the following: developer, publication year, country, study design, subjects, sample size, analytical methods, assessment tools, number of symptom clusters, and type of symptom clusters.

## Results

### Study characteristics

**Figure 1:**
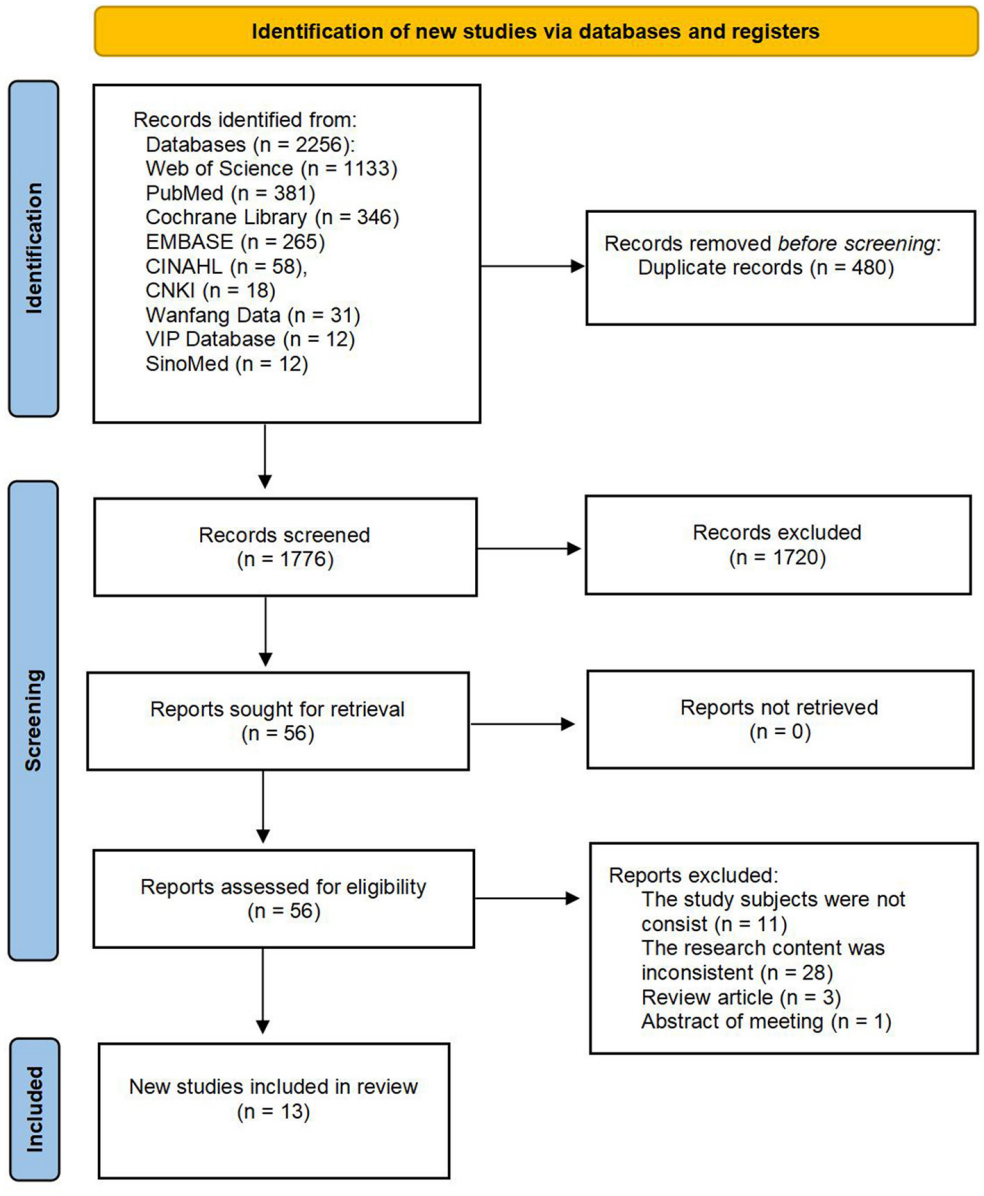
Flow chart of literature selection.

The initialsearch retrieved a total of 2,256 articles from the following databases. Two independent reviewers screened these articles and reached consistent results based on the predefined inclusion and exclusion criteria, ultimately including 13 articles (see Figure 1) (18, 22–33).

All included articles were published within the past 8 years, reflecting the growing attention and importance given to symptom clusters by researchers (see Table 1). These studies were primarily from China (*n* = 7) (22, 24–26, 28, 32, 33) and the United States (*n* = 3) (18, 23, 29), with additional studies from Canada (*n* = 1) (31), Norway (*n* = 1) (27), and the United Kingdom (*n* = 1) (30). The studies included 12 quantitative studies (18, 23–33) and one qualitative study (22). Specifically, seven were cross-sectional studies (18, 24–26, 28, 31, 32), and six were longitudinal studies (22, 23, 27, 29, 30, 33). The subjects of these studies included patients with IBD, UC, and CD.

**Table 1:** Characteristic information of literature.

**References**	**Country**	**Study design**	**Subjects**	**Sample**	**Analytical**
Conley et al. (18)	USA	Cross-sectional study	IBD	5,296	Latent class analysis
Conley et al. (23)	USA	Longitudinal study	IBD	5,296	Latent class analysis
Perler et al. (29)	USA	Longitudinal study	IBD	233	Principal component analysis
Sexton et al. (31)	Canada	Cross-sectional study	IBD	267	Exploratory factor analysis
Gu et al. (25)	China	Cross-sectional study	IBD	148	Exploratory factor analysis
Gu (24)	China	Cross-sectional study	IBD	207	Descriptive analysis
Guan et al. (26)	China	Cross-sectional study	UC	120	Exploratory factor analysis
Xu et al. (32)	China	Cross-sectional study	IBD	83	Descriptive analysis
Liu et al. (28)	China	Cross-sectional study	IBD	270	Descriptive analysis
Chen (22)	China	Longitudinal qualitative study	CD	18	Semi-structured interview
Johansen et al. (27)	Norway	Longitudinal study	IBD	350	Principal component analysis
Riggot et al. (30)	UK	Longitudinal study	IBD	692	Latent class analysis
Zhijia et al. (33)	China	Longitudinal study	IBD	206	Descriptive analysis

### The types and characteristics of symptom clusters

A total of 29 symptom clusters were identified across the 13 included studies, as detailed in Table 2. These clusters varied considerably in nomenclature, with overlapping symptom compositions frequently observed across different studies. Some clusters were labeled according to symptom type (e.g., abdominal, bowel, or psychological clusters), whereas others were defined by symptom burden (e.g., low vs. high symptom burden) or trajectory (e.g., stable vs. rapid decline groups).

**Table 2:** The types of symptom clusters.

**References**	**Number**	**Type of symptom clusters**
Conley et al. (18)	4	Low symptom burden, high symptom burden, physical symptom cluster, psychological symptom cluster
Conley et al. (23)	4	Low symptom burden, high symptom burden, physical symptom cluster, psychological symptom cluster
Perler et al. (29)	4	Bowel frequency and abdominal discomfort symptom cluster, systemic/extraintesinal symptom cluster, anorectal symptom cluster, abdominal symptom cluster, incontinence and flatulence symptom cluster
Sexton et al. (31)	5	Bowel symptom cluster, abdominal symptom cluster, fatigue symptom cluster, bowel complications symptom cluster, systemic complications symptom cluster
Gu et al. (25)	5	Abdominal symptom cluster, bowel complications symptom cluster, nutritional symptom cluster, physical symptom cluster, psychological symptom cluster
Gu (24)	5	Abdominal symptom cluster, bowel complications symptom cluster, nutritional symptom cluster, physical symptom cluster, psychological symptom cluster
Guan et al. (26)	2	Intestinal function related sympot cluster, negative state symptom cluster
Xu et al. (32)	5	Abdominal symptom cluster, bowel complications symptom cluster, nutritional symptom cluster, physical symptom cluster, psychological symptom cluster
Liu et al. (28)	5	Abdominal symptom cluster, bowel complications symptom cluster, nutritional symptom cluster, physical symptom cluster, psychological symptom cluster
Chen (22)	6	Mouth ulcers–loss of appetite–weight loss, abdominal pain–sleep disorders–fatigue–loss of appetite–weight loss, abdominal pain–urgency of stool–diarrhea–perianal pain–fatigue, bloating–abdominal pain–nausea–vomiting, abdominal pain–anxiety–diarrhea, anxiety–depression–fear–despair–sleep disorders
Johansen et al. (27)	3	Psychological symptom cluster, impaired energy cluster cluster, physical symptom cluster
Riggott et al. (30)	3	Below- average gastrointestinal and psychological symptoms, average levels of gastrointestinal and psychological symptoms, the highest levels of both gastrointestinal and psychological symptoms
Zhijia et al. (33)	3	Moderate symptom cluster stable decline group, high symptom cluster-rapid decline group, stable symptom cluster-stable trend group

Given this overlap and inconsistency, we further synthesized the reported clusters into five broader categories to facilitate interpretation: gastrointestinal/physical, psychological, systemic/fatigue, nutritional/appetite-related, and mixed/trajectory-related. Representative terms used in the original studies for each category are summarized in Table 3.

**Table 3:** Core symptom cluster categories and representative terms in IBD.

**Core symptom cluster**	**Number of studies**	**Representative terms used**
Gastrointestinal/Physical	11	Physical symptom cluster; Abdominal symptom cluster; Bowel symptom cluster; Bowel frequency & abdominal discomfort symptom cluster; Anorectal symptom cluster; Incontinence & flatulence symptom cluster; Bowel complications symptom cluster; Intestinal function–related symptom cluster; Bloating–abdominal pain–nausea–vomiting.
Psychological	9	Psychological symptom cluster; Negative state symptom cluster; Anxiety–depression–fear–despair–sleep disorders.
Systemic/fatigue	4	Fatigue symptom cluster; Systemic complications symptom cluster; Impaired energy cluster.
Nutritional/appetite-related	5	Nutritional symptom cluster; Mouth ulcers–loss of appetite–weight loss; Systemic/extraintestinal symptom cluster (incl. appetite loss).
Mixed/trajectory-related	5	Low symptom burden; High symptom burden; Combined gastrointestinal & psychological clusters (below-average/average/high levels); Stable–stable trend/Moderate–stable decline/High–rapid decline groups; Abdominal pain–anxiety–diarrhea; Abdominal pain–urgency of stool–diarrhea–perianal pain–fatigue; Abdominal pain–sleep disorders–fatigue–loss of appetite–weight loss.

### Assessment tools for symptom clusters

This study included 13 papers, which utilized a diverse range of assessment tools. Seven primary assessment tools were employed: PROMIS, symptom inventory, IBDSI, SCS-IBD, psychological and gastrointestinal symptom measures, UC symptom scale, and MSAS (see Table 4). The SCS-IBD was the most frequently used (*n* = 5) (24, 25, 28, 32, 33). The SCS-IBD and the MSAS assessed symptom clusters based on occurrence frequency, severity, and distress. The PROMIS was used in two studies (18, 23), the IBDSI in one study (31), self-administered scales in two studies (29, 30), the UC symptom scale in one study (22), and the MSAS in one study (27).

**Table 4:** The information of assessment tools.

**Assessment tools**	**Number of studies**	**Studies**
PROMIS^a^	2	(18, 23)
Symptom inventory	1	(29)
IBDSI^b^	1	(31)
SCS-IBD^c^	5	(24, 25, 28, 32, 33)
Psychological and gastrointestinal symptom measures	1	(30)
UC symptom scale	1	(26)
MSAS^d^	1	(27)

a Patient Reported Outcome Measurement Information System;

b Inflammatory Bowel Disease of Symptom Index;

c Symptom Cluster Scale for Inflammatory Bowel Disease;

d Memorial Symptom Assessment Scale.

### Factors associated with symptom clusters in IBD

Seven studies identified demographic, clinical, psychological, and biological factors associated with symptom clusters in IBD (see Table 5). High-burden and psychological clusters were linked to female sex, younger age, smoking, corticosteroid use, and active disease, while gastrointestinal clusters were associated with ulcerative colitis, disease severity, and low hemoglobin. Psychological clusters correlated with anxiety, depression, and reduced quality of life, and fatigue-dominant clusters were related to vitamin D deficiency. Longitudinal studies further showed that cluster transitions predicted adverse outcomes such as high flares and hospitalization.

**Table 5:** Variables and outcomes associated with symptom clusters in IBD.

**Category**	**Variable**	**Association**	**Symptom cluster (s)**	**Outcome (if reported)**	**References**
Demographic factors	Female	Associated with increased risk	High-burden; Psychological	NR	(18)
	Younger age	Associated with increased risk	High-burden	NR	(18)
	Older age	Associated with increased risk	GI cluster; Psychological (mixed)	Higher flare rate; Increased hospitalization	(30)
Lifestyle factors	Smoking	Associated with increased risk	High-burden; Psychological	NR	(18)
Clinical status	Remission	Associated with decreased risk	All clusters	NR	(18)
	Transition active → remission	Predicted transition from high-burden to low-burden/psychological	Low-burden; Psychological	NR	(23)
	Disease severity	Associated with increased risk	GI cluster	Associated with higher disease activity and lower QoL	(26)
	Disease subtype (UC)	Associated with increased risk	GI cluster; Psychological (mixed)	Higher flare rate; Increased hospitalization; Lower QoL	(26, 30)
	Low hemoglobin	Associated with increased risk	GI cluster	Lower QoL	(26)
	Vitamin D deficiency	Associated with increased risk	Fatigue cluster	NR	(27)
Treatment-related factors	Corticosteroid use	Associated with increased risk	High-burden; GI; Psychological (mixed)	Increased hospitalization	(18, 30)
Psychological/psychosocial factors	Anxiety	Associated with increased risk	Psychological cluster	Lower QoL	(26)
	Depression	Associated with increased risk	Psychological cluster	Lower QoL	(26)
	Maladaptive coping style	Associated with increased risk	Psychological cluster	Reduced QoL	(28)
	Low QoL domains (social, emotional)	Associated with increased risk	Psychological cluster	Reduced QoL	(28)
	Illness perception and emotional appraisal	Associated with symptom cluster trajectories	Psychological/mixed clusters	NR	(33)

NR, Not reported by study authors; QoL, Quality of Life.

### Characteristics of symptom cluster changes

#### Acute phase

During the acute phase of IBD, particularly at the initial onset, patients often experience a sudden and intense manifestation of symptoms, which is associated with a strong inflammatory response at this stage. Symptom clusters tend to be more pronounced during this period. A longitudinal study (23) found that most IBD patients with a heavy symptom burden at baseline tended to maintain this condition at 6- and 12-month follow-ups, forming a high-burden symptom cluster primarily characterized by persistent symptoms such as pain, fatigue, sleep disturbances, depression, and anxiety. Although some patients experience a reduction in symptom burden as the disease transitions from the active to the remission phase, improvement in symptom clusters is not always significant (27). This indicates that even when disease activity decreases, patients in the acute phase may still endure a considerable symptom burden. Furthermore, symptom clusters in the acute phase often involve a combination of abdominal symptoms (e.g., diarrhea, bloating), systemic symptoms (e.g., pain, fatigue), and psychological symptoms (e.g., depression, anxiety) (31), reflecting a substantial burden on both physiological and psychological levels. These findings underscore the need to not only control inflammatory responses during the acute phase but also to address the comprehensive symptom burden—particularly the long-term impact on mental health.

#### Remission phase

In the remission phase of IBD, patients' clinical symptoms exhibit a characteristic shift, and symptom clusters become more stable. Research has shown that although abdominal and intestinal symptoms are often effectively managed during this stage, psychological symptoms tend to persist. This shift in symptom pattern suggests that the remission of physiological symptoms in IBD does not necessarily coincide with the resolution of psychological symptoms. Overall, as the disease enters remission, the symptom burden generally decreases, with notable improvements in systemic, abdominal, and intestinal symptom clusters. It is important to note, however, that some patients experience a transformation in symptom cluster types—from those dominated by systemic symptoms to those characterized mainly by psychological symptoms. A study by Conley et al. (23) reported that as patients transitioned from the active to the remission phase, many shifted from high-burden symptom clusters to either low-burden or psychological symptom clusters. Nonetheless, not all patients achieve complete symptom resolution during remission. The persistence of certain symptoms, especially psychological ones, highlights the long-term impact of IBD on patients' quality of life. Therefore, even when physiological symptoms are effectively controlled during remission, ongoing attention to the psychological wellbeing of patients remains essential.

## Discussion

This scoping review systematically synthesizes evidence from 13 studies on symptom cluster types, assessment instruments, associated factors, and the dynamic trajectories of symptom clusters in patients with inflammatory bowel disease (IBD). The findings provide a comprehensive framework to guide future research directions and inform clinical management strategies.

### Symptom cluster types and nomenclature

Our review revealed substantial inconsistencies in the naming and composition of symptom clusters in inflammatory bowel disease (IBD). Two main approaches were identified: some studies defined clusters descriptively by symptom type (e.g., abdominal or psychological clusters), whereas others labeled them by dominant symptoms (e.g., anxiety–depression–sleep disturbance). Disagreement was most apparent for symptoms with lower factor loadings, which were variably classified across studies. Fatigue illustrates this challenge (34): while Conley et al. (18) grouped it with sleep disturbance in a physical cluster, Johansen et al. (27) described it as an impaired energy cluster, and Gu (24) classified it as psychosomatic. Given its multifactorial and poorly understood etiology, fatigue requires further investigation to clarify its biological and psychological associations.

To synthesize existing findings, we propose five provisional cluster categories: (1) gastrointestinal/physical, (2) psychological, (3) systemic/fatigue, (4) nutritional/appetite-related, and (5) mixed/trajectory-related. This framework integrates diverse nomenclature into a coherent structure while acknowledging areas of overlap and divergence. Gastrointestinal/physical clusters were consistently observed, but their composition varied, ranging from abdominal pain and diarrhea to anorectal complications and extraintestinal manifestations. Psychological clusters almost universally included anxiety, depression, and sleep disturbance, though fatigue was inconsistently classified. The systemic/fatigue cluster showed the greatest heterogeneity: Perler et al. (29) defined it broadly to include musculoskeletal pain, ocular symptoms, dizziness, and insomnia, whereas Gu (24) restricted it to dermatologic, oral, and ocular manifestations. Despite these differences, both reflect extraintestinal involvement consistent with Chinese expert consensus (35). Nutritional/appetite-related clusters, though less frequently reported, highlight the impact of appetite loss, weight change, and oral lesions. Finally, mixed/trajectory-related clusters capture longitudinal variability, distinguishing stable from worsening symptom trajectories and offering prognostic insights.

Overall, our review shows considerable inconsistency in the naming and composition of IBD symptom clusters, which largely reflects limited exploration of the underlying mechanisms. This lack of clarity may affect how researchers classify symptoms into clusters and interpret their significance. Future studies should therefore aim to clarify the core constructs of symptom clusters, investigate their biological and psychosocial mechanisms, and use longitudinal and multicenter designs to test their stability across populations. In parallel, standardized analytic approaches are needed to improve reproducibility and enable consistent identification of cluster categories across studies.

### Assessment tools for IBD symptom clusters: strengths and limitations

In studies of symptom clusters among patients with inflammatory bowel disease (IBD), both single- and multi-symptom assessment tools have been applied. Single-symptom instruments facilitate focused measurement but risk overlooking interactions between co-occurring symptoms. In contrast, multidimensional tools such as the MSAS and PROMIS can capture symptom trajectories, but they often lack IBD-specific sensitivity.

Among the 13 studies reviewed, seven instruments were identified, with the SCS-IBD and PROMIS most frequently applied. The SCS-IBD, validated primarily in Chinese cohorts, provides reliable multidimensional assessment of symptom frequency, severity, and distress (24, 36). However, the absence of multicenter validation limits its broader generalizability. PROMIS, developed within the NIH framework, demonstrates strong psychometric properties and facilitates comparisons across chronic diseases (37). Yet, in IBD populations it fails to capture key disease-specific symptoms such as diarrhea, urgency, and abdominal distension (38, 39), which may lead to systematic underestimation of symptom burden. The MSAS, despite strong reliability in oncology (40), has not been adapted to the gastrointestinal context and may therefore overlook critical IBD manifestations. The IBDSI and its short form (IBDSI-SF) were designed for repeated monitoring of IBD symptoms and have demonstrated robust psychometric validity (31), with reported associations to inflammatory biomarkers (41). However, as they rely solely on patient-reported outcomes, their scores demonstrate only partial concordance with objective disease activity measures such as endoscopy or biochemical markers. Two studies (29, 30) employed self-developed questionnaires, but their limited validation raises concerns about reproducibility and comparability, underscoring the broader challenges of standardizing symptom assessment in IBD research.

Taken together, existing tools provide valuable insights but face persistent challenges, including limited disease specificity, reliance on subjective reporting, and uncertain applicability across disease phases. Future research should therefore prioritize: (i) developing IBD-specific, multidimensional, and clinically feasible instruments; (ii) establishing dynamic evaluation systems integrating patient-reported outcomes with objective markers such as endoscopy and fecal calprotectin; and (iii) clarifying optimal assessment intervals and validating tool performance across both active and remission phases. Addressing these issues will support the development of a more comprehensive and standardized framework for IBD symptom cluster assessment, thereby enhancing clinical decision-making, treatment evaluation, and the comparability of research findings across studies.

### Associated factors of symptom clusters in IBD

Across the included studies, a range of demographic, clinical, and treatment-related variables were found to influence symptom cluster membership. High-burden and psychological clusters were more common among women, younger patients, and smokers (18), whereas gastrointestinal clusters were linked to ulcerative colitis, greater disease severity, and low hemoglobin (26). Fatigue-dominant clusters were associated with vitamin D deficiency (27). Psychological comorbidities, including anxiety, depression, and maladaptive coping styles, consistently amplified symptom burden and were associated with reduced quality of life (26, 28). Treatment exposures, particularly corticosteroid use, were repeatedly associated with high-burden or mixed clusters (18, 30), raising questions about potential iatrogenic contributions to symptom clustering. Importantly, several studies suggested that cluster membership can shift over time, with transitions predicting adverse outcomes such as increased flare rates, hospitalization, and surgery (23, 30). Despite these insights, most studies employed cross-sectional designs, relied heavily on self-reported data, and rarely incorporated objective inflammatory markers (e.g., CRP, fecal calprotectin). Moreover, few analyses stratified findings by disease subtype. Perler et al. (29) systematically distinguished UC and CD, and further stratified by disease extent in UC and disease location in CD, highlighting important heterogeneity in symptom presentation. In their inception cohort, UC most commonly presented with bloody bowel movements and diarrhea, whereas CD more often presented with fatigue and abdominal pain, underscoring differences in dominant symptom domains. Guan et al. (26), focusing specifically on UC, identified two major clusters (intestinal function-related and negative state clusters) and demonstrated that disease extent significantly influenced the intestinal function cluster, suggesting that even within a single subtype, anatomical distribution can shape clustering. Rimmer et al. (42), in a large UK triage study including over 400 patients with UC or CD, similarly reported that rectal bleeding and urgency were more common in UC, whereas abdominal pain and fatigue predominated in CD, but symptom clusters were not analyzed separately by subtype.

In summary, the available evidence indicates that symptom cluster membership in IBD is determined by a multifactorial interplay of demographic, clinical, psychological, treatment-related, and disease-specific variables. Preliminary evidence also suggests that gastrointestinal clusters appear to be more prominent in UC, whereas systemic, nutritional, and psychological manifestations may be more characteristic of CD, with disease extent or location potentially modifying these patterns. Future research should explicitly stratify findings by disease subtype and extent, and employ longitudinal, biomarker-integrated, and multicenter designs to clarify these determinants and their clinical implications.

### Management strategies for dynamic changes of symptom clusters in IBD

Findings from six longitudinal studies included in this review indicate that symptom clusters in IBD patients are not static but instead evolve dynamically with disease progression. Therefore, individualized management strategies tailored to different disease stages are essential to enhance the scientific basis and effectiveness of interventions. (1) Acute Phase: Management should focus on controlling inflammation and alleviating physical symptoms. During acute IBD flare-ups, symptom burden increases significantly (43). Abdominal symptom clusters—such as diarrhea, bloating, and abdominal pain—are the most common and prominent, often appearing in multidimensional co-occurrence. Studies have shown (29, 44) that diarrhea and bloating are core symptoms within this cluster, exhibiting high persistence and stability. Additionally, patients in the acute phase often experience altered gut microbiota diversity, which may exacerbate symptoms and contribute to the severity of extraintestinal manifestations. Some studies (45–47) have also found that symptom fluctuations closely align with disease activity, and a portion of patients present with significant psychological symptoms such as anxiety and depression during this phase. Therefore, management at this stage should prioritize the relief of physical symptoms and effective inflammation control, while also focusing on early identification and intervention for emotional distress. (2) Remission Phase: Emphasis should be placed on the identification and intervention of psychological symptoms. Although gastrointestinal symptoms improve significantly during remission, some patients continue to experience psychological distress. Conley et al. (23) through a longitudinal study, found that some patients remained in the “psychological symptom group” during remission, characterized by persistent anxiety, depression, and sleep disturbances. Zhijia et al. (33) observed that even after achieving clinical remission with biological agents (e.g., infliximab), some patients still exhibited moderate to severe symptom clusters, predominantly psychological in nature. These findings suggest that clinical remission does not equate to complete symptom relief, and the residual psychological symptoms tend to be insidious and enduring. Thus, ongoing psychological assessment and intervention should be a key component of remission-phase management. (3) Long-Term Stable Phase: Attention should be given to dynamic symptom monitoring and individualized adjustments. During the relatively stable long-term management phase, patients display substantial individual variation in symptom expression (48). Some experience marked symptom relief, while others may continue to suffer from persistent abdominal or psychological discomfort (49). Therefore, regular assessment of symptom cluster changes is necessary to dynamically capture patients' subjective experiences and disease evolution. Based on these findings, intervention strategies should be flexibly adjusted to achieve precise, long-term individualized care.

This review has several strengths, including its comprehensive scope and synthesis of findings across diverse study designs. Nonetheless, some limitations should be acknowledged. First, the search was restricted to English and Chinese publications and excluded gray literature, which may have introduced selection bias. Second, substantial heterogeneity in study populations, assessment instruments, and analytic methods limited comparability, and most tools were not specifically validated for IBD, raising concerns about measurement validity. Third, the majority of included studies were cross-sectional and rarely incorporated objective biomarkers, making it difficult to examine causal relationships, cluster–cluster interactions, or links with biological disease activity. Finally, few studies stratified findings by disease subtype, which limits conclusions about potential differences between UC and CD. As a scoping review, no formal quality appraisal or meta-analysis was undertaken, precluding causal inference or quantitative effect estimation. These limitations highlight the need for longitudinal, biomarker-integrated, and methodologically standardized research to advance the science of symptom clusters in IBD.

## Conclusion

In summary, this scoping review synthesized evidence from 13 studies and identified five core symptom clusters in patients with inflammatory bowel disease (IBD): gastrointestinal, psychological, fatigue, impaired energy, and pain. These clusters were associated with demographic, clinical, psychological, and lifestyle factors, and in some cases linked to adverse outcomes such as poorer quality of life, increased disease activity, and greater healthcare utilization. However, existing studies relied on heterogeneous and non-IBD–specific instruments, and few assessed longitudinal trajectories, biological correlates, or interactions between clusters. Importantly, most did not stratify by disease subtype, although limited evidence suggests that UC appears to be characterized primarily by gastrointestinal clusters, whereas CD more often involves systemic, nutritional, or psychological clusters. Advancing this field will require biomarker-integrated, methodologically standardized, and longitudinal research to clarify the mechanisms of symptom clustering and inform tailored interventions. Ultimately, a better understanding of symptom clusters may help clinicians provide more precise symptom management and improve patient-centered outcomes in IBD.
